# Self-rated physical health predicts mortality in aging persons beyond objective health risks

**DOI:** 10.1038/s41598-023-46882-7

**Published:** 2023-11-09

**Authors:** Anna C. Reinwarth, Felix S. Wicke, Nora Hettich, Mareike Ernst, Danielle Otten, Elmar Brähler, Philipp S. Wild, Thomas Münzel, Jochem König, Karl J. Lackner, Norbert Pfeiffer, Manfred E. Beutel

**Affiliations:** 1 Department of Psychosomatic Medicine and Psychotherapy, University Medical Center of the Johannes Gutenberg-University Mainz, Mainz, Germany; 2 Department of Psychiatry and Psychotherapy, University Medical Center of the Johannes Gutenberg-University Mainz, Mainz, Germany; 3 Department of Clinical Psychology, Psychotherapy, and Psychoanalysis, Institute of Psychology, University of Klagenfurt, Klagenfurt am Wörthersee, Austria; 4 Department of Cardiology - Cardiology I, University Medical Center of the Johannes Gutenberg-University Mainz, Mainz, Germany; 5 Institute of Clinical Chemistry and Laboratory Medicine, University Medical Center of the Johannes Gutenberg-University Mainz, Mainz, Germany; 6 Department of Ophthalmology, University Medical Center of the Johannes Gutenberg-University Mainz, Mainz, Germany; 7 Institute of Medical Biostatistics, Epidemiology and Informatics, University Medical Center of the Johannes Gutenberg-University Mainz, Mainz, Germany; 8 Preventive Cardiology and Preventive Medicine - Department of Cardiology, University Medical Center of the Johannes Gutenberg-University Mainz, Mainz, Germany; 9 Center for Thrombosis and Hemostasis, University Medical Center of the Johannes Gutenberg-University Mainz, Mainz, Germany; 10 Institute of Molecular Biology (IMB), Mainz, Germany; 11 German Center for Cardiovascular Research (DZHK), Partner Site Rhine-Main, Mainz, Germany; 12 Department of Psychiatry and Psychotherapy, Medical Faculty, University of Leipzig, Leipzig, Germany

**Keywords:** Psychology, Risk factors

## Abstract

Previous studies on self-rated health and mortality have usually not differentiated between physical and mental health, respectively have not considered physical diseases. This study aims to determine self-rated physical and mental health from middle to old age, examine associations with mortality adjusted for objective risk factors and assess effect modification by gender. In a large population-based sample (*N* = 14,993 at baseline), self-rated physical and mental health were rated separately by a single-item. Associations to mortality were modelled by Cox regressions, adjusting for potential confounding variables. Most participants rated their physical (79.4%), resp. mental health (82.3%) as good. Poor self-rated physical health was lowest in the youngest group (19.6%, age 35–44), and highest in midlife (29.1%, age 55–64). Poor self-rated mental health was lowest among the oldest (18.5%), and highest from 45 to 54 years (29.3%). Poor self-rated physical, but not mental health was predictive of mortality when adjusting for objective risk factors. Male gender and poor self-rated physical health interacted (RERI 0.43 95%-CI 0.02–0.85). Self-rated physical health was best in the youngest and worst in the midlife group, this pattern was reversed regarding self-rated mental health. Poor self-rated physical, but not mental health was predictive of mortality, adjusting for objective risk factors. It was more strongly predictive of mortality in men than in women. Poor subjective physical health ratings, should be taken seriously as an unfavorable prognostic sign, particularly in men.

## Introduction

Self-rated health is the subjective individual’s assessment of their health^1^. It has become an easily-assessed and important indicator of healthy aging^2,3^, reflecting not only the presence of illnesses, complaints, and functional limitations, but also personal well-being. Most likely, subjective and objective aspects of health are combined from the perceptual framework of the individual respondent^4^. Thus, self-rated health has been postulated to reflect health aspects relevant to mortality that are not covered by objective indicators^4^.

Since the 1950s, self-rated health^5^ has been included as a global indicator of population health, mostly in midlife and aging populations, in predicting future health-related events and the use of medical care^4^. To assess self-rated health, single item questions have been formulated, e.g. "In general, how would you rate your health today?" Responses have been grouped into very good or good, moderate, poor or very poor^6^. In a large (*N* = 23,906) German representative survey from 2014/15, 68,2% rated their general health as good or very good^5^. Self-rated health varies with a multitude of socioeconomic factors. Good or very good self-rated health was highest in the youngest (18–29 years) and lowest in the elderly group (65 + years)^7^. Poor self-rated health has been reported by women (vs. men)^4^, individuals with low education^8^ and income, and those with somatic^7^ and mental illness^9^. Overall, poor self-rated health was associated with higher mortality^10–12^. In their meta-analysis, DeSalvo et al.^13^ reported two-fold higher mortality risk for persons with poor, compared to excellent self-rated health. Benjamins, et al.^14^ found that self-rated health was predictive to different degrees of mortality due to the major medical illnesses, but not for deaths by accident, homicide or suicide. Predictivity decreased, however, associations with mortality were maintained when adjusting for co-morbidity, depression, subclinical illness or functional status^15,16^.

The association between self-rated health and mortality may also depend on gender and age. Sex-mediated temporal differences exist across almost all major medical diseases^17^. Lin et al.^18^ found that men were at a greater risk for mortality despite reporting better health and functional performance than women. However, some studies have reported stronger associations in females than in males^19,20^. The US National Health Interview Survey^21^ found that poor self-rated health affected the survival of younger more compared to older individuals.

Usually, studies have inquired about health in general, without distinguishing the major dimensions of physical and mental health. Self-rated mental health, assessed similarly to self-rated physical health, has been associated consistently and to a moderate degree with indicators of mental morbidity. Poor self-rated mental health was associated with poor self-rated general health, physical health problems, and health service utilization^22^. However, analysing the effect of self-rated mental and physical health on mortality, Sajjad, et al.^23^ found that self-rated mental health is not predictive of mortality among participants from the Rotterdam Study, when including sociodemographic, major chronic physical diseases, functional status, and mental health indicators.

While previous German studies on this topic mostly reported demographic and health behaviour data e.g.^5^, only a few studies include objective assessments of medical illnesses to determine if self-rated health contains information that is not entirely reflected in underlying medical illnesses. For instance, Heidrich et al.^24^ estimated relative hazards for all-causes mortality and cardiovascular disease according to global self-rated health in middle-aged men and women followed from 1984 to 1995 taking medical illnesses and cardiovascular risk factors into account. Findings suggested that poor global self-rated health increases the risk of mortality compared to good self-rated health among men but not women. However, it is currently unclear whether self-rated physical health or self-rated mental health both accounts for the association with mortality in men and women within a recent large cohort study of the German population when medical illnesses are included in the models. As Sajjad et al.^23^ showed that only self-rated physical, but not mental health is predictive of mortality, after adjusting for relevant covariates, we assumed that is important to assess the effect of self-rated physical and mental health on mortality separately.

This longitudinal study assesses the impact of self-rated health on mortality in a large, representative sample of the German population distinguishing the two major dimensions of physical and mental health. Specifically, we aimed to:

1. determine the prevalence of good vs. poor self-rated physical and mental health from middle to old age
2. examine the associations of self-rated physical and mental health with mortality while adjusting for objectively assessed risk factors
3. assess effect modification by gender on the associations between self-rated health and mortality.

## Results

### Participants and baseline characteristics

Table 1 presents sociodemographic and health characteristics of the total sample (*N* = 14,993) according to poor vs. good self-rated physical, resp. mental health.

**Table 1:** Baseline participant characteristics (stratified by self-rated physical and mental health).

	Overall	Self-rated physical health			Self-rated mental health		
		Good	Bad	*p*-value	Good	Bad	*p*-value
n	15,010	11,922	3069		12,356	2637	
Age (M, SD)	55.01 (11.10)	54.69 (11.19)	56.21 (10.70)	< 0.001	55.13 (11.22)	54.43 (10.54)	0.004
Sex (male) (%)	7584 (50.5)	6189 (51.9)	1384 (45.1)	< 0.001	6594 (53.4)	980 (37.2)	< 0.001
Married (yes) (%)	11,144 (74.3)	9034 (75.8)	2105 (68.6)	< 0.001	9447 (76.5)	1694 (64.2)	< 0.001
Living alone (yes) (%)	2828 (18.9)	2059 (17.3)	767 (25.0)	< 0.001	2058 (16.7)	769 (29.2)	< 0.001
Socioeconomic status (M, SD)	12.89 (4.48)	13.22 (4.45)	11.58 (4.35)	< 0.001	13.09 (4.50)	11.94 (4.28)	< 0.001
PHQ-9 > = 10 (yes) (%)	1133 (7.7)	467 (4.0)	666 (22.5)	< 0.001	311 (2.6)	821 (32.1)	< 0.001
GAD-2 > = 3 (yes) (%)	966 (6.6)	470 (4.0)	496 (16.9)	< 0.001	275 (2.3)	690 (27.2)	< 0.001
Loneliness (yes) (%)	2495 (17.0)	1589 (13.6)	905 (30.7)	< 0.001	1503 (12.4)	990 (39.0)	< 0.001
Smoking (pack-years) (M, SD)	4.89 (11.37)	4.42 (10.58)	6.70 (13.91)	< 0.001	4.71 (11.11)	5.70 (12.50)	< 0.001
Body mass index (M, SD)	27.38 (5.02)	26.88 (4.60)	29.29 (6.05)	< 0.001	27.28 (4.88)	27.85 (5.62)	< 0.001
Social support (BS6) (M, SD)	20.45 (3.68)	20.76 (3.46)	19.23 (4.23)	< 0.001	20.85 (3.40)	18.55 (4.31)	< 0.001
Chronic diseases (%)				< 0.001			< 0.001
0	5918 (39.4)	5071 (42.5)	834 (27.2)		4934 (39.9)	972 (36.9)	
1	5553 (37.0)	4476 (37.5)	1072 (34.9)		4607 (37.3)	941 (35.7)	
2	2190 (14.6)	1586 (13.3)	603 (19.6)		1774 (14.4)	416 (15.8)	
3	865 (5.8)	543 (4.6)	322 (10.5)		679 (5.5)	186 (7.1)	
> = 4	484 (3.2)	246 (2.1)	238 (7.8)		362 (2.9)	122 (4.6)	
Death = 1 (%)	1254 (8.4)	851 (7.1)	398 (13.0)	< 0.001	1027 (8.3)	222 (8.4)	0.887

Participant characteristics are shown as mean values and standard deviations or as percentages and absolute numbers. Reported percentages related to the total sample (*N* = 14,993), *M* =   mean, *SD* = standard deviation, SES = socioeconomic status, BMI = body mass index.

Overall, about 79.4% rated their physical health and 82.3% their mental health as good. Mean age was slightly higher in poor physical and lower in poor mental health. Female gender, living alone, being unmarried, 2nd generation migration status, a lower SES were related to poor physical and mental health. The same was true for depression, anxiety, loneliness, smoking, BMI, and number of chronic physical diseases. However, rates of mental distress and loneliness were higher in poor mental health, and chronic physical diseases were more frequent in poor physical health.

During a mean follow-up period of 11.2 years (median 11.5 years), 1249 (8.4%) participants died, 13% in those with poor vs. 7.1% in those with good physical health. Mental health was not associated with mortality (8.4% in poor vs. 8.3% in good mental health). Mortality was almost twice as high in men (10.8%) than in women (5.9%).

**Figure 1:**
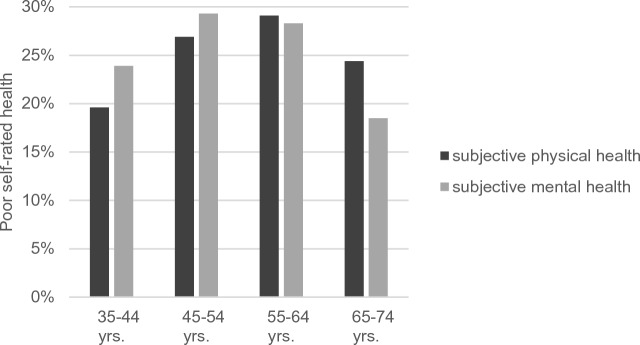
Proportions of poor physical and mental health in the total sample (*N* = 14,993), stratified by age groups.

Figure 1 presents the proportions of poor physical and mental health in the total sample of 14,993 participants across the age span.

As Fig. 1 illustrates, poor physical health was reported only by 19.6% in the youngest age group and was more often reported (29.1%) in the age group from 55 to 64 years, but there was no further increase at 65–74 years. Poor mental health was highest in the age group from 45 to 54 years, but was lowest among the oldest group.

**Figure 2:**
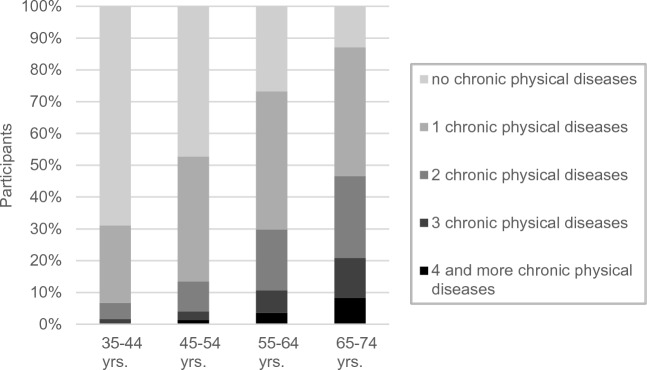
Numbers of chronic physical illnesses in the total sample (*N* = 14,993), stratified by age groups.

Figure 2 presents the numbers of chronic physical illnesses over the age span.

As the figure shows, chronic diseases were already present in a minority of 31.1% of the age group from 35 to 44 years and were present in the majority of 87.1% at the age of 65–74 years. The number of chronic physical diseases and particularly the proportions of multiple chronic illnesses increased strongly with age.

### Self-rated health and mortality

Table 2 shows the association between self-rated physical and mental health and mortality for the total sample.

**Table 2:** Results of the cox proportional hazards regression models: Prediction of all-cause mortality on self-rated physical and mental health at baseline, adjusting for confounders.

Self-rated physical health				Self-rated mental health		
	HR	95% CI	*p*-value	HR	95% CI	*p-*value
Model 1, n = 14,991; events = 1249; concordance = 0.78				Model 1, *n* = 14,993; events = 1249; concordance = 0.78		
Self-rated health (poor)	1.94	1.72–2.19	< 0.001	1.30	1.12–1.50	0.001
Gender (men)	1.93	1.72–2.17	< 0.001	1.89	1.68–2.12	< 0.001
Age	1.11	1.10–1.12	< 0.001	1.11	1.10–1.12	< 0.001
Model 2, n = 14;238: events = 1172; concordance = 0.81				Model 2, *n* = 14,241; events = 1172; concordance = 0.81		
Self-rated health (poor)	1.35	1.19–1.54	< 0.001	1.08	0.92–1.26	0.352
Gender (men)	1.75	1.54–2.00	< 0.001	1.73	1.52–1.98	< 0.001
Age	1.09	1.09–1.10	< 0.001	1.09	1.08–1.10	< 0.001
BMI (< 18.5)	2.26	1.16–4.40	0.017	2.28	1.17–4.44	0.016
BMI (25–30)	0.84	0.72–0.99	0.032	0.85	0.72–0.99	0.035
BMI (30–35)	0.91	0.76–1.08	0.279	0.93	0.78–1.10	0.39
BMI (35–40)	0.95	0.74–1.22	0.677	0.99	0.77–1.27	0.912
BMI (> = 40)	1.65	1.21–2.26	0.002	1.77	1.30–2.42	< 0.001
Chronic diseases = 1	1.29	1.06–1.57	0.011	1.29	1.06–1.57	0.011
Chronic diseases = 2	1.89	1.53–2.33	< 0.001	1.94	1.57–2.39	< 0.001
Chronic diseases = 3	2.60	2.06–3.29	< 0.001	2.77	2.20–3.49	< 0.001
Chronic diseases > = 4	3.59	2.81–4.59	< 0.001	3.89	3.05–4.95	< 0.001
Log (smoking (pack-years))	1.29	1.22–1.35	< 0.001	1.30	1.23–1.36	< 0.001
Smoking (packyears = zero)	0.96	0.83–1.12	0.633	0.97	0.84–1.13	0.706
Socioeconomic status	0.96	0.94–0.97	< 0.001	0.95	0.94–0.97	< 0.001

Model 1 adjusting for age and gender, Model 2 adjusting additionally for subclinical health indicators, number of chronic physical disease, smoking, and socioeconomic status. *HR* = hazard ratio; Concordance = Goodness-of-fit-index; BMI = body mass index.

Self-rated physical health was predictive of mortality when age and gender were taken into account; male gender and higher age were predictive of mortality (model 1). In model 2, poor self-rated physical health was still predictive of mortality with an increase of 26% (HR 1.26; 95% CI 1.03–1.55) after controlling for objective risk factors. Male gender and higher age were still predictive of mortality, along with a low (< 18.5 kg/m^2^) and a high BMI (> = 40), whereas mild obesity (25–30) was negatively associated with mortality. The presence of chronic physical diseases was strongly predictive of mortality, the hazard ratios increased with the number of chronic physical diseases. Four and more chronic physical diseases were associated with a 3.58 fold increase in mortality (HR 3.58; 95% CI 2.8–4.58) compared to no chronic physical disease. Higher SES was associated with lower mortality.

Self-rated mental health was only predictive of mortality when age and gender were taken into account; male gender and higher age were predictive of mortality (model 1). In the fully adjusted model (model 2), however, poor self-rated mental health was no longer associated with higher mortality. For the other predictors results were similar to the physical health model.

Table 3 shows the results of effect modification analyses, specifically how the effect of self-rated physical health on mortality is modified by gender.

**Table 3:** Results of effect modification analyses: Interaction of self-rated physical health and gender.

	Self-rated physical health good	Self-rated physical health poor	Effect of SPH within the strata of gender
	HR [95% CI]*	HR [95% CI]*	HR [95% CI]*
Model 1: adjusted for age			
Women	1 [Reference]	1.66 [1.37, 2.03]	1.66 [1.37, 2.03]
Men	1.78 [1.54, 2.05]	3.79 [3.2, 4.49]	2.13 [1.83, 2.47]
Multiplicative scale	1.28 [1, 1.64]		
RERI	1.35 [0.78, 1.91]		
Model 2: adjusted for age, BMI, chronic disease count, smoking, and SES			
Women	1 [Reference]	1.26 [1.03, 1.55]	1.26 [1.03, 1.55]
Men	1.69 [1.45, 1.97]	2.39 [1.98, 2.89]	1.41 [1.2, 1.66]
Multiplicative scale	1.12 [0.86, 1.44]		
RERI	0.43 [0.02, 0.85]		

Effect modification by gender was assessed on additive and multiplicative scales. Effect modification on the additive scale was calculated by the relative excess risk of interaction (RERI) with 95%-confidence intervals, a value significantly different from 0 indicating effect modification. Effect modification on the multiplicative scale was measured by multiplicative interaction terms in Cox models, a value significantly different from 1 indicating effect modification. *HR* = Hazard ratio, BMI = Body mass index, SES = Socioeconomic status.

In analyses adjusted for age only, the relative risk of death was highest for men with poor self-rated physical health (HR: 3.79, 95%-CI 3.2–4.49, as compared to women with good self-rated physical health). In the fully adjusted analysis, poor self-rated physical health was more strongly predictive of mortality in men (HR 1.41; 95-CI 1.2–1.66) than in women (HR 1.26; 95%-CI 1.03–1.55). Again, the relative risk of death was highest for men with poor self-rated physical health (HR: 2.39, 95%-CI 1.98–2.89, as compared to women with good self-rated physical health) and this interaction was superadditive on the additive scale (RERI 0.43 95%-CI 0.02–0.85), i.e. men with poor self-rated health had even higher mortality than what would have been expected from the association of being male and having a poor self-rated health status with mortality alone. Interaction on the multiplicative scale was not statistically significant (interaction term HR 1.12; 95%-CI 0.86–1.44). We also tested for a modifying effect of gender on self-rated mental health. No significant effect was observed (detailed results not shown).

## Discussion

The present work aimed to determine the prevalence of good vs. poor self-rated physical and mental health from middle to old age, examine the associations of self-rated physical and mental health with mortality while adjusting for objectively assessed risk factors, and assess effect modification by gender on the associations between self-rated health and mortality. We found that the great majority of participants reported good physical and mental health across the age span from 35 to 74 years. About 20.5% reported poor self-rated physical, respectively self-rated mental health (17.6%). The distribution of these two ratings differed across the age range. The youngest age group reported the lowest poor physical health, whereas poor mental health was the least frequent in the oldest age group. Poor physical health was most frequently reported by 29.1% in the age group from 55 to 64 years. Poor mental health was most frequent (29.3%) in the age group from 45 to 54 years. This is consistent with so-called midlife crises which have been described in men and women around their 50s^25^. Better physical and mental health was rated by men, married participants, and those with a higher SES. It was lower in those living alone and suffering from depression and anxiety symptoms. The presence of chronic illnesses was more strongly associated with self-rated physical health.

The presence and numbers of chronic physical diseases increased steadily with age and were highest in the oldest group of age 65 and higher; however, this was not accompanied by a decrease of self-rated physical health. Thus, the relationship between objective health indicators and self-rated physical health appears to shift over the age span. A possible explanation for this finding is the theory of response shift, a change in respondents’ frame of references^26,27^. Therefore, we assume that aging individuals adjust to an increasing number of chronic physical diseases by a change of their internal frames of references and maintain their self-rated health. Ernst, et al.^28^ explored subjective health appraisal and mental distress of cancer survivors and showed that years since diagnosis were negatively related to mental health disorder. Furthermore, self-rated health improved with more time since diagnosis. Alternatively, there may be a bias due to survival effect. E.g. those in poor self-rated health die earlier. As expected, only poor self-rated physical health was associated with a higher rate of mortality. The association decreased when objective health information (BMI, number of chronic physical diseases, and smoking) and sociodemographic status were entered into the regression model, all of which had an impact on mortality. Still, the association between self-rated physical health and mortality was retained. Poor self-rated mental health was only associated with mortality when age and gender, but no objective health factors were considered. This may reflect the negative impact of physical ill health on mental health^28,29^.

As in previous studies, men had higher mortality during the observation period of more than 11 years on average, but rated their subjective physical and mental health better than women^5^. When we performed effect modification analyses, the increase of mortality in poor vs. good self-rated health, however, was higher in men than in women. As delineated by Jylhä^4^, self-rated health assessments probably result from combining subjective and objective aspects of health in the individual conceptual framework. As our data indicate, there may also be a shift in expectations. E.g., in middle adulthood, the absence of chronic physical diseases may indicate good self-rated health. In the elderly, however, one’s health may be perceived as good in the presence of chronic physical diseases which are perceived as expectable in the age reached and well- controlled (e.g., in coronary heart disease). Not surprisingly, the association between self-rated physical health and mortality declined when demographic characteristics, chronic physical diseases, and health behaviour were taken into account.

Knowledge or awareness of one’s objective health is an important part of self-rated health. An intriguing, yet poorly understood finding remains that poor self-rated physical health has an additive effect on objective indicators of poor health in predicting mortality. Self-rated health can be used as part of an older individual's health evaluation when screening for future adverse outcomes, particularly in men, and also as an important indicator of healthy aging. Overall, the majority report good subjective health. Self-rated physical health is best in the youngest and self-rated mental health is best in the oldest group. The number of chronic physical diseases is positively associated with age. However, age-related increases of chronic physical diseases are not reflected in poorer self-rated physical health in the elderly. Thus, additional factors, e.g. their more favourable mental health may play a protective role, whereas higher functional impairments may adversely affect self-rated physical health. Poor vs. good self-rated physical, but not mental health predicts mortality, even when adjusting for sociodemographic characteristics, chronic physical diseases objective health, health behaviour and socioeconomic status, with larger effect in men compared to women.

We assessed self-rated physical and mental health at baseline. However, self-rated health may have changed throughout the long-term follow-up. Chronic physical diseases were also assessed based on self-report and objective findings at baseline. Thus, we cannot preclude that a certain proportion of participants were unaware of their chronic physical diseases. While we included major chronic physical diseases, our list also was not comprehensive. Participants may have taken into account additional chronic physical diseases, and they may also have considered functional status, bodily complaints, and sensations which may indicate somatic dysfunction (e.g. fatigue and inflammatory processes; Jylhä^4^). While we adjusted for BMI, major chronic physical diseases, smoking, and SES, other health behaviour variables such as alcohol consumption and physical activity were not included due to missing data. Based on cross-sectional data, we cannot resolve the question, how self-rated physical and mental health evolve over the life span. However, their trajectories differ between the age groups. Consistent with Jokela et al.^30^, self-rated physical health was best in the youngest group, whereas self-rated mental health was best in the oldest group. Due to the use of cross-sectional data of self-rated health, we were not able to account for potential cohort effects. The focus of this paper was on mortality, and we did not test the hypothesis that poor self-rated mental health may be predictive of subsequent depression^31^.We used all-cause mortality as outcome, therefore we cannot assume any prediction on cause-specific mortality. Further studies should integrate cause-specific mortality data into the analysis to aim at a better understanding of underlying mechanisms.

The majority of men and women aged 35–74 years rate their subjective physical and mental health as good. The trajectory of self-rated health across the age span indicates that increasing rates of chronic physical diseases are reflected in higher ratings of poor health. However, this does not apply to the elderly group, who suffers from most chronic physical diseases. Thus, individuals may adjust to a growing burden of chronic physical diseases; the absence of chronic physical disease is not a prerequisite for good self-rated health in an aging population. Obviously, self-rated health captures relevant health information beyond objective health indicators, and may be relevant to mortality. Future analyses will therefore identify determinants of self-rated health. In order to better understand the meaning of self-rated health, future analyses will assess the course of physical and mental health, their determinants and potential interaction over the life span.

## Methods

### Study design and participants

The Gutenberg Health Study (GHS) is an ongoing population-based, prospective, observational single-center cohort study in the Rhine-Main region located in western Mid-Germany^32^. Myocardial infarction and cardiovascular death were defined as the primary endpoints of the study. Additional endpoints were mortality and diseases of the eye, the immune system, cancer, and mental health. The study protocol was approved by the ethics committee of the Medical Chamber of Rhineland-Palatinate and the local and federal data safety commissioners. The sample of the GHS was drawn at random from the local registries of the city of Mainz and the district of Mainz-Bingen. The random sample was stratified 1:1 for gender and residence and in equal strata across age decades. According to the study protocol, inclusion criteria were age 35–74, and exclusion criteria were insufficient knowledge of the German language, and physical or mental inability to visit the study center for study investigations. Written informed consent was obtained from each participant before their inclusion in the study, according to the tenets of the Declaration of Helsinki. The present study was based on the baseline examination of 14,993 participants (2007–2012). An overview of number of missing values for most important variables can be found as Supplementary Table S1.

### Measures

All-cause mortality from baseline assessment onwards was the primary outcome. Mortality updates were performed by quarterly queries to the registry offices and the mortality registry Rhineland-Palatinate. Death certificates with the exact date of death were acquired for death reviews.

Self-rated physical and mental health as independent variables were assessed by the two items “How would you rate your current physical health?” and “How would you rate your current mental health?” Response options ranged from very good to bad. We dichotomized self-rated health status: good self-rated health combined the responses 1 = very good and 2 = good; poor self-rated health combined the responses 3 = not so good and 4 = bad.

In a computer-assisted personal interview, participants were asked whether they had ever received a definite diagnosis of any of the major chronic physical diseases by a physician, cardiovascular diseases (myocardial infarction, coronary artery disease, stroke, peripheral artery disease, atrial fibrillation, heart failure), cancer, migraine, pulmonary diseases (asthma, COPD), hypertension and diabetes. Hypertension was defined by intake of antihypertensive drugs or by a systolic blood pressure of at least 140 mmHg (or diastolic blood pressure of at least 90 mmHg), measured at rest in a sitting position on the right arm in our study center. Diabetes was defined in individuals with a definite diagnosis of diabetes by a physician or a blood glucose level of ≥ 126 mg/dl in the baseline examination after an overnight fast of at least 8 h or a blood glucose level of > 200 mg/dl after a fasting period of 8 h. Numbers of diseases were summarized.

Mental health measures included depression, generalized anxiety and loneliness. Depression was assessed with the depression module of the Patient Health Questionnaire PHQ-9^33^. Participants rated the frequency of each of the 9 diagnostic criteria of major depression over the past two weeks on a Likert scale (0 = not at all, 1 = several days, 2 = more than half the days, 3 = nearly every day). Answers were summed to a score of 0 to 27 points. Depression was defined by 10 or more points. The two-item Generalized Anxiety Disorder Screener GAD-2^34^, was used to assess generalized anxiety. Participants rated “Feeling nervous, anxious or on edge” and “Not being able to stop or control worrying” on the same scale as depression. A sum score of 3 or more was used as cut-off to define generalized anxiety. Loneliness was assessed by the validated single-item “I am frequently alone /have few contacts”^35,36^. Response options ranged from 0 = “no, does not apply”, 1 = “yes, it applies, but I do not suffer from it”, 2 = “yes, it applies, and I suffer slightly”, 3 = “yes, it applies, and I suffer moderately”, 4 = “yes, it applies, and I suffer strongly”. In line with previous research a binary variable combining responses 0 and 1 to indicate “no loneliness”, and 2–4 to indicate “loneliness” was used^36^.

Bodyweight and height were measured in the study center using standardized procedures. Participants were assigned to the categories of BMI < 18.5, 18.5 < 25, 25 < 30, 30 < 35, and BMI >  = 35, defined by bodyweight in kg/(height in m)^2^. Smoking was assessed by self-report. We calculated pack-years of smoking as number of cigarettes smoked per day divided by 20 (a pack) and multiplied by the number of years smoked. Due to the skewness of the variable, it was log-transformed for inclusion in the regression models. Smoking was set to „1 “ for those with zero pack-years and to „0 “ for those with > 0 pack-years of smoking.

Sociodemographic characteristics were assessed as self-report and included age in years, gender as male and female, married [no/yes], living alone [no/yes]. Combining data of education, profession and income, we defined socioeconomic status (SES) according to Lampert and Kroll^37^ ranging from 3 (lowest) to 27 (highest) SES.

Descriptive characteristics of the analysed sample were reported as absolute numbers and percentages for categorical variables and as means with standard deviations for continuous variables.

To investigate the effect of good vs. poor self-rated physical and mental health on all-cause mortality and to adjust for confounders, cox proportional hazards regression models were calculated. Self-rated physical and mental health were analyzed in separate models. In a first step, we first tested all sociodemographic variables, subclinical health indicators, number of chronic physical diseases, mental health indicators, health behavior indicators, and socioeconomic status with respect to their uni-/bivariate associations with self-rated physical and mental health. We chose to sequentially add blocks of confounding variables, with Model 1 adjusting for age and gender, Model 2 adjusting additionally for subclinical health indicators (BMI), number of chronic physical disease, smoking as indicator for health behaviour and socioeconomic status.

Effect modification by gender was assessed both on additive and multiplicative scales. Relative excess risk of interaction (RERI) with 95%-confidence intervals, as described by Li and Chambless^38^, was calculated as a measure for effect modification on an additive scale, with a value significantly different from 0 indicating effect modification. Multiplicative interaction terms in Cox models were used as a measure for effect modification on a multiplicative scale, with a value significantly different from 1 indicating effect modification^39,40^.

All analyses were done using R version 4.0.3^41^ with the packages survival^42^, tableOne^43^, psych^44^, epiR^45^ and interactionR^46^.

The founding source had no role in the design, conduct or reporting of the present study or in the decision to submit the manuscript for publication.

### Supplementary Information

Supplementary Information.

## Data Availability

The datasets analysed during the current study are not publicly available. The written informed consent of the study participants is not suitable for public access of the data and this concept was not approved by the local data protection officer and ethics committee. But access to data at the local database in accordance with the ethics vote is offered upon reasonable request at any time. Interested researchers make their requests of the Principal Investigator of the GHS (Philipp.Wild@unimedizin-mainz.de).

## References

[1] Viljanen A (2021). Subjective and objective health predicting mortality and institutionalization: an 18-year population-based follow-up study among community-dwelling Finnish older adults. BMC Geriatr..

[2] Diener E, Pressman SD, Hunter J, Delgadillo-Chase D (2017). If, why, and when subjective well-being influences health, and future needed research. Appl. Psychol. Health Well Being.

[3] Krause L, Lampert T (2015). Relation between overweight/obesity and self-rated health among adolescents in Germany. Do socio-economic status and type of school have an impact on that relation?. Int. J. Environ. Res. Public Health.

[4] Jylhä M (2009). What is self-rated health and why does it predict mortality? Towards a unified conceptual model. Soc. Sci. Med..

[5] Lampert T, Schmidtke C, Borgmann L-S (2018). Subjektive Gesundheit bei Erwachsenen in Deutschland. J. Health Monit..

[6] de Bruin A, Picavet HS, Nossikov A (1996). Health interview surveys. Towards international harmonization of methods and instruments. WHO Reg. Publ. Eur. Ser..

[7] Zhang L, Bi X, Ding Z (2021). Health lifestyles and Chinese oldest-old’s subjective well-being-evidence from a latent class analysis. BMC Geriatr..

[8] Byun M, Kim E, Ahn H (2021). Factors contributing to poor self-rated health in older adults with lower income. Healthcare (Basel).

[9] Liu S, Qiao Y, Wu Y, Shen Y, Ke C (2021). The longitudinal relation between depressive symptoms and change in self-rated health: A nationwide cohort study. J. Psychiatr. Res..

[10] Bamia C (2017). Self-rated health and all-cause and cause-specific mortality of older adults. Individual data meta-analysis of prospective cohort studies participating in the CHANCES Consortium. Maturitas.

[11] Heistaro S, Jousilahti P, Lahelma E, Vartiainen E, Puska P (2001). Self rated health and mortality: A long term prospective study in eastern Finland. J. Epidemiol. Commun. Health.

[12] Wuorela M (2020). Self-rated health and objective health status as predictors of all-cause mortality among older people: A prospective study with a 5-, 10-, and 27-year follow-up. BMC Geriatr..

[13] DeSalvo KB, Bloser N, Reynolds K, He J, Muntner P (2006). Mortality prediction with a single general self-rated health question. J. Gen. Intern. Med..

[14] Benjamins MR, Hummer RA, Eberstein IW, Nam CB (2004). Self-reported health and adult mortality risk: an analysis of cause-specific mortality. Soc. Sci. Med..

[15] Murata C, Kondo T, Tamakoshi K, Yatsuya H, Toyoshima H (2006). Determinants of self-rated health: Could health status explain the association between self-rated health and mortality?. Arch. Gerontol. Geriatr..

[16] Fried LP (1989). Risk factors for 5-year mortality in older adults: The Cardiovascular Health Study. Jama.

[17] Westergaard D, Moseley P, Sorup FKH, Baldi P, Brunak S (2019). Population-wide analysis of differences in disease progression patterns in men and women. Nat. Commun..

[18] Lin MH (2022). Age and sex differences in associations between self-reported health, physical function, mental function and mortality. Arch. Gerontol. Geriatr..

[19] Leinonen R, Heikkinen E, Jylhä M (2001). Predictors of decline in self-assessments of health among older people—a 5-year longitudinal study. Social. Sci. Med..

[20] Szybalska A (2018). Self-rated health and its association with all-cause mortality of older adults in Poland: The PolSenior project. Arch. Gerontol. Geriatr..

[21] Cho H (2022). Estimating life expectancy adjusted by self-rated health status in the United States: National health interview survey linked to the mortality. BMC Public Health.

[22] Ahmad F, Jhajj AK, Stewart DE, Burghardt M, Bierman AS (2004). Single item measures of self-rated mental health: a scoping review. BMC Health Serv. Res..

[23] Sajjad A (2017). Subjective measures of health and all-cause mortality–the Rotterdam study. Psychol. Med..

[24] Heidrich J, Liese AD, Löwel H, Keil U (2002). Self-rated health and its relation to all-cause and cardiovascular mortality in southern Germany. Results from the MONICA Augsburg cohort study 1984–1995. Ann. Epidemiol..

[25] Beutel ME, Glaesmer H, Wiltink J, Marian H, Brahler E (2010). Life satisfaction, anxiety, depression and resilience across the life span of men. Aging Male.

[26] Sprangers MA, Schwartz CE (1999). Integrating response shift into health-related quality of life research: A theoretical model. Social. Sci. Med..

[27] Schwartz, C. E. & Sprangers, M. A. G (Eds.). *Adaptation to changing health: Response shift in quality-of-life research*. American Psychological Association. https://psycnet.apa.org/doi/10.1037/10382-000 (2000).

[28] Ernst M (2019). Linking cancer and mental health in men and women in a representative community sample. J. Psychosom. Res..

[29] Otten D (2022). Depressive symptoms predict the incidence of common chronic diseases in women and men in a representative community sample. Psychol. Med..

[30] Jokela M, Batty GD, Kivimaki M (2013). Ageing and the prevalence and treatment of mental health problems. Psychol. Med..

[31] Perna L (2020). Subjective mental health, incidence of depressive symptoms in later life, and the role of epigenetics: Results from two longitudinal cohort studies. Transl. Psychiatry.

[32] Wild PS (2012). The Gutenberg health study. Bundesgesundheitsblatt Gesundheitsforschung Gesundheitsschutz.

[33] Löwe B, Kroenke K, Herzog W, Gräfe K (2004). Measuring depression outcome with a brief self-report instrument: Sensitivity to change of the Patient Health Questionnaire (PHQ-9). J. Affect. Disorders.

[34] Kroenke K, Spitzer RL, Williams JB, Monahan PO, Löwe B (2007). Anxiety disorders in primary care: Prevalence, impairment, comorbidity, and detection. Ann. Intern. Med..

[35] Beutel ME (2017). Loneliness in the general population: Prevalence, determinants and relations to mental health. BMC Psychiatry.

[36] Reinwarth AC, Ernst M, Krakau L, Brahler E, Beutel ME (2023). Screening for loneliness in representative population samples: Validation of a single-item measure. PLoS ONE.

[37] Lampert T, Kroll L, Müters S (2013). Messung des sozioökonomischen Status in der Studie zur Gesundheit Erwachsener in Deutschland (DEGS1). Bundesgesundheitsbl.

[38] Li R, Chambless L (2007). Test for additive interaction in proportional hazards models. Ann. Epidemiol..

[39] VanderWeele TJ, Knol MJ (2014). A tutorial on interaction. Epidemiol. Methods.

[40] Knol MJ, VanderWeele TJ (2012). Recommendations for presenting analyses of effect modification and interacstion. Int. J. Epidemiol..

[41] R: A Language and Environment for Statistical Computing. (2020).

[42] Therneau TM, Grambsch PM (2000). Modeling Survival Data: Extending the Cox Model.

[43] tableone: Create ’Table 1’ to Describe Baseline Characteristics with or without Propensity Score Weights (2020).

[44] psych: Procedures for Psychological, Psychometric, and Personality Research. (2021).

[45] epiR: Tools for the Analysis of Epidemiological Data. (2021).

[46] interactionR: Full Reporting of Interaction Analyses. (2020).

