# Supplementary material for: Self-rated physical health predicts mortality in aging persons beyond objective health risks
Source: Sci Rep. 2023 Nov 9;13:19531. doi: 10.1038/s41598-023-46882-7 (PMC10636131; doi:10.1038/s41598-023-46882-7)
Supplement: Supplementary file 1 — Supplementary Information. [file 41598_2023_46882_MOESM1_ESM.docx]

| Variable | n (%) of missing at baseline |
| --- | --- |
| Self-rated physical health | 19 (0.12) |
| Self-rated mental health | 17 (0.11) |
| gender (men) | 0 (0) |
| age | 0 (0) |
| BMI | 11 (0.07) |
| pack-years (cigarettes) | 704 (4.69) |
| socioeconomic status | 98 (0.65) |

**Supplementary Table S1.** Missings for main variables at baseline (*N*=15,010)
